# Low-ammonia niche of ammonia-oxidizing archaea in rotating biological contactors of a municipal wastewater treatment plant

**DOI:** 10.1111/j.1462-2920.2012.02786.x

**Published:** 2012-09

**Authors:** Laura A Sauder, Francien Peterse, Stefan Schouten, Josh D Neufeld

**Affiliations:** 1 Department of Biology, University of Waterloo200 University Avenue West, Waterloo, Ontario, Canada N2L 3G1; 2 Department of Marine Organic Biogeochemistry, NIOZ Royal Netherlands Institute for Sea ResearchTexel, The Netherlands

## Abstract

The first step of nitrification is catalysed by both ammonia-oxidizing bacteria (AOB) and archaea (AOA), but physicochemical controls on the relative abundance and function of these two groups are not yet fully understood, especially in freshwater environments. This study investigated ammonia-oxidizing populations in nitrifying rotating biological contactors (RBCs) from a municipal wastewater treatment plant. Individual RBC stages are arranged in series, with nitrification at each stage creating an ammonia gradient along the flowpath. This RBC system provides a valuable experimental system for testing the hypothesis that ammonia concentration determines the relative abundance of AOA and AOB. The results demonstrate that AOA increased as ammonium decreased across the RBC flowpath, as indicated by qPCR for thaumarchaeal *amoA* and 16S rRNA genes, and core lipid (CL) and intact polar lipid (IPL) crenarchaeol abundances. Overall, there was a negative logarithmic relationship (*R*^2^ = 0.51) between ammonium concentration and the relative abundance of AOA *amoA* genes. A single AOA population was detected in the RBC biofilms; this phylotype shared low *amoA* and 16S rRNA gene homology with existing AOA cultures and enrichments. These results provide evidence that ammonia availability influences the relative abundances of AOA and AOB, and that AOA are abundant in some municipal wastewater treatment systems.

## Introduction

Ammonia is a metabolic waste product that threatens aquatic ecosystems with toxicity, oxygen depletion and algal blooms. A primary objective of wastewater treatment is to prevent these adverse environmental impacts by removing ammonia from wastewater prior to discharge into receiving waters. Ammonia removal in wastewater treatment is accomplished through nitrification, a microbially mediated process in which ammonia is oxidized to nitrite and subsequently to nitrate. Wastewater treatment plants (WWTPs) may release nitrate-rich effluents, or nitrate may be reduced to dinitrogen gas via anaerobic ammonia oxidation (anammox) or denitrification prior to effluent discharge. Until recently, only specific members of the *Beta*- and *Gammaproteobacteria*, known as ammonia-oxidizing bacteria (AOB), were believed to be capable of catalysing the process of ammonia oxidation. Understanding of nitrification in natural environments changed with the discovery that members of the newly proposed phylum *Thaumarchaeota* (Brochier-Armanet *et al*., 2008; Spang *et al*., 2010) are capable of ammonia oxidation (Könneke *et al*., 2005), and that ammonia-oxidizing archaea (AOA) are ubiquitous in natural environments (Francis *et al*., 2005; Prosser and Nicol, 2008). In fact, AOA outnumber AOB in a variety of environments including soils (Leininger *et al*., 2006), marine habitats (Wuchter *et al*., 2006; Beman *et al*., 2008; De Corte *et al*., 2008) and estuarine sediments (Beman and Francis, 2006). However, AOB are numerically dominant in some sampled environments, including the San Francisco Bay Estuary (Mosier and Francis, 2008), as well as industrial (Limpiyakorn *et al*., 2010) and municipal (Wells *et al*., 2009) WWTPs.

Recent studies suggest that ammonia availability influences niche separation of AOA and AOB. Kinetic studies of the AOA isolate *Nitrosopumilus maritimus* demonstrate an exceptionally high substrate affinity for ammonia; *N. maritimus* has a half saturation constant (*K*_m_) of 0.133 µM total ammonia (Martens-Habbena *et al*., 2009), which is approximately two orders of magnitude lower than that of *Nitrosomonas oligotropha*, for which reported *K*_m_ values range from 30 to 75 µM total ammonia (Stehr *et al*., 1995). As a result of this high substrate affinity andthe high relative abundance of AOA in oligotrophic environments, ammonia availability has been suggested to be an important factor in determining niche partitioning of AOA and AOB (Erguder *et al*., 2009; Schleper, 2010). Studies of soil AOA support this observation by demonstrating that AOB are numerically and metabolically dominant in ammonium-amended soils (Jia and Conrad, 2009; Di *et al*., 2010; Taylor *et al*., 2010; Verhamme *et al*., 2011). However, few studies have examined the role of ammonia availability in determining relative abundances of AOA and AOB in freshwater environments, especially those in engineered water treatment systems.

**Fig. 1:**
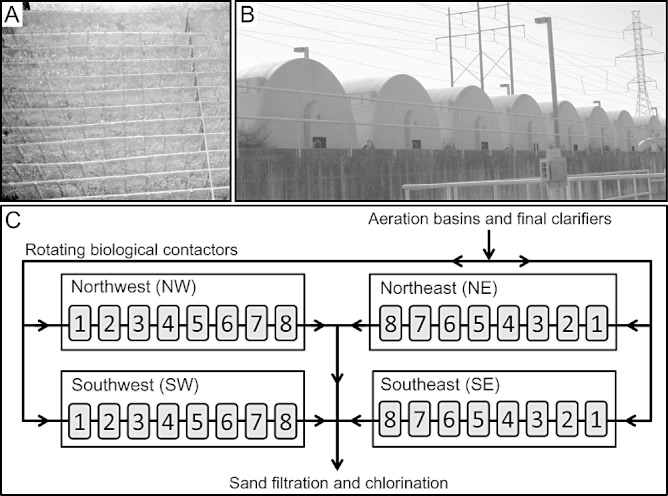
Outline of the sampling site in Guelph, Ontario. Internal medium of an RBC (A), an external view of a full RBC treatment train (B) and a schematic of the RBC arrangement in the Guelph WWTP (C).

This study utilized existing nitrification infrastructure in a municipal WWTP in Guelph, Ontario, Canada to investigate the effect of ammonia concentrations on the abundance and diversity of prokaryotic ammonia-oxidizing communities. The Guelph WWTP features a tertiary treatment system of rotating biological contactors (RBCs), which are nitrification bioreactors comprised of panels of corrugated polymeric medium (Fig. 1A) attached to a central rotating shaft and partially submerged in wastewater. Eight individual RBC stages are arranged in series (Fig. 1B and C), with nitrification at each stage creating an ammonia gradient across the RBC flowpath. Although previous studies have detected AOB in nitrifying RBCs through PCR-based methods and microscopy (Egli *et al*., 2003; Pynaert *et al*., 2003; Jang *et al*., 2005), no study has investigated the occurrence, abundance or diversity of AOA in nitrifying RBCs. We hypothesized that the abundance of AOA would increase across the RBC flowpath, as wastewater becomes increasingly depleted of ammonium.

## Results

### Water chemistry

**Fig. 2:**
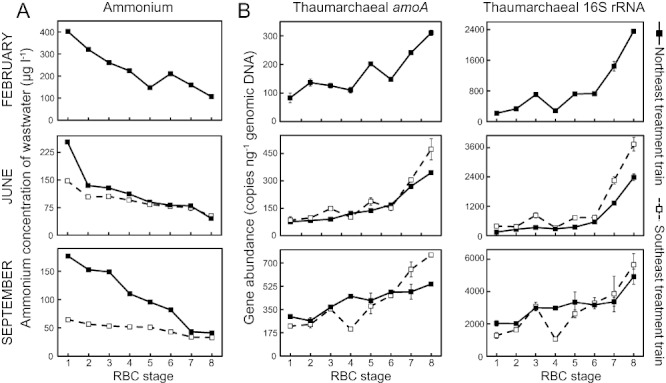
Ammonium concentrations of wastewater (A) and thaumarchaeal *amoA* and 16S rRNA gene abundances in associated biofilm samples (B) across RBC flowpaths. Error bars represent standard deviations based on technical duplicates; error bars that are not seen are contained within the symbols, except for ammonium concentrations in the February NE treatment train, for which duplicate measurements are not available.

In all sampling seasons and both the Northeast (NE) and Southeast (SE) treatment trains (see *Experimental procedures* for sampling details), ammonium decreased alongthe RBC flowpaths (Fig. 2A and Table S1). Overall, wastewater ammonium concentrations were highest in February and lowest in September, and were consistently higher in the NE treatment train than the SE train. Nitrite decreased across RBC flowpaths in patterns similar to ammonium, and nitrite concentrations were always relatively low (i.e. < 400 µg l^−1^, Table S1). In contrast, nitrate concentrations were always high (15–30 mg l^−1^), and measured nitrate levels did not change in a predictable manner across individual RBC flowpaths. For all RBC stages in all seasons, the pH varied within a narrow range of 7.2 to 7.6. Other parameters, such as temperature and dissolved organic carbon (DOC), varied little across a given RBC flowpath but showed seasonal differences. Dissolved oxygen (dO_2_) in this aerated system was always greater than 6 mg l^−1^, and increased by ≤ 2 mg l^−1^ across a given RBC flowpath.

### Gene abundances

Thaumarchaeal *amoA* and 16S rRNA gene sequences were detected in all RBC stages from all seasons. In each RBC train sampled, AOA *amoA* gene abundance in genomic DNA extracts obtained from biofilm increased across the RBC flowpath (Fig. 2B). In addition, thaumarchaeal 16S rRNA gene abundance increased across the flowpath, in patterns congruent with archaeal *amoA* genes (Fig. 2B). For both thaumarchaeal *amoA* and 16S rRNA genes, abundance varied by season; gene abundances were highest in September and lowest in February (Fig. 2B). In addition, in both June and September, gene abundances were higher in the SE treatment train than the NE train.

**Fig. 3:**
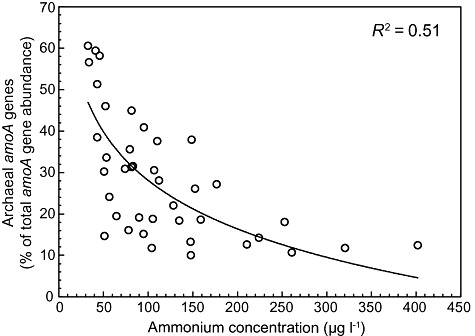
Ammonium concentrations of RBC-associated wastewater and relative abundance of archaeal *amoA* genes (as a per cent of total archaeal and bacterial *amoA* genes per nanogram of genomic DNA) in corresponding RBC biofilm.

Bacterial *amoA* genes were detected in biofilm extracts from all RBC stages. In contrast to AOA-associated genes, bacterial *amoA* gene abundance did not show predictable or consistent patterns between or across RBC trains when analysed independently (Fig. S1A). In addition, general bacterial 16S rRNA gene abundances (measured as a control gene) were consistent across all RBC stages, regardless of treatment train or season (Fig. S1B). When biofilm and associated wastewater from all RBC stages (i.e. from all sampling times and treatment trains) were considered together, the relative abundance of AOA *amoA* genes (as a proportion of total *amoA* genes) comprised 10–61% of the total ammonia-oxidizing community (Fig. 3). The relative abundance of AOA *amoA* genes demonstrated a negative logarithmic trend with ammonium concentration (*R*^2^ = 0.51; Fig. 3). Furthermore, Spearman's rank correlation coefficients were negative and statistically significant for influent ammonia concentrations with both the relative abundance of AOA *amoA* genes (i.e. as a proportion of total *amoA* genes; *r* = −0.6887, *P* < 0.0001) as well as independent AOA *amoA* gene abundances (*r* = −0.6088, *P* < 0.0001).

### Lipid abundances

**Fig. 4:**
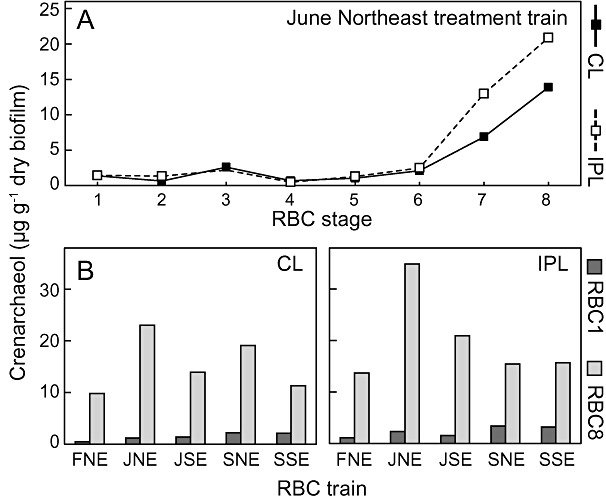
Lipid analysis. CL- and IPL-derived crenarchaeol abundance in biofilm across the June NE RBC flowpath (A) and core and IPL-derived crenarchaeol abundances for RBC 1 and 8 biofilm for all seasons and treatment trains (B). Letters F, J and S denote February, June and September respectively.

In addition to quantification of key genes, concentrations of crenarchaeol, the characteristic membrane lipid of ammonia-oxidizing thaumarchaea (Pitcher *et al*., 2011a and references therein) were determined both as core lipids (CLs) as well as intact polar lipids (IPLs). Core lipids are assumed to represent fossilized (i.e. dead) biomass, while IPLs are biomarkers indicative of viable microbial cells (Sturt *et al*., 2004; Biddle *et al*., 2006; Pitcher *et al*., 2009; 2011b). Crenarchaeol in biofilm samples from all RBC stages of the June NE treatment train was quantified; both CL- and IPL-derived crenarchaeol increased across the RBC flowpath (Fig. 4A). For all other RBC trains, lipid analyses were performed for RBCs 1 and 8 only. In all cases, biofilm samples from RBC 8 yielded higher CL and IPL crenarchaeol abundances than RBC 1 (Fig. 4B).

### Gene diversity across RBC flowpath

**Fig. 5:**
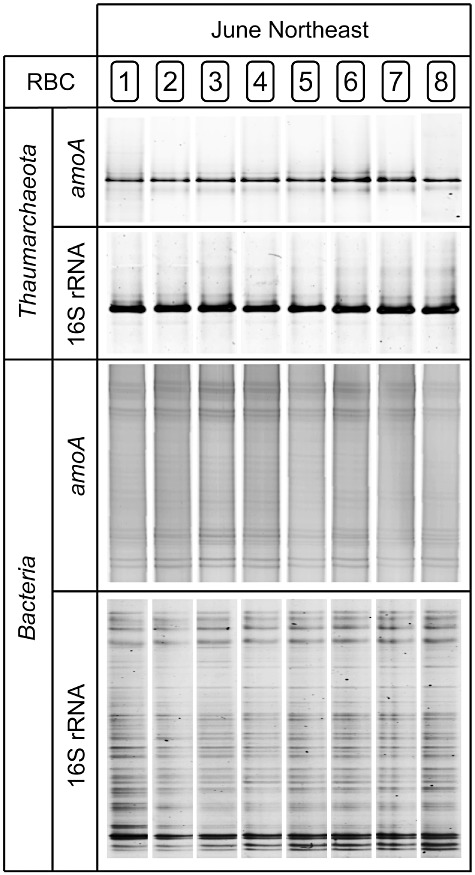
DGGE fingerprints for bacterial and thaumarchaeal *amoA* and 16S rRNA genes across eight serial RBC stages. Data shown were obtained from the NE treatment train, sampled in June 2010. Data from all RBC trains are shown in Fig. S1.

For all genes analysed, communities were highly similar across all RBC stages of a given flowpath (Figs 5 and S2). Denaturing gradient gel electrophoresis (DGGE) profiles for thaumarchaeal *amoA* and 16S rRNA genes revealed simple patterns and low diversity; one dominant band was observed for both archaeal *amoA* and thaumarchaeal 16S rRNA (Fig. 5). For both thaumarchaeal genes analysed, diversity and community composition were nearly identical across all RBC stages sampled in all seasons (Figs 5 and S2). Bacterial *amoA* DGGE profiles were more complex, and patterns were similar across a given flowpath and between seasons (Figs 5 and S2). General bacterial 16S rRNA gene fingerprints were complex and highly similar, both across RBCs of a given treatment train (Fig. 5) and between treatment trains and seasons (Fig. S2).

**Fig. 6:**
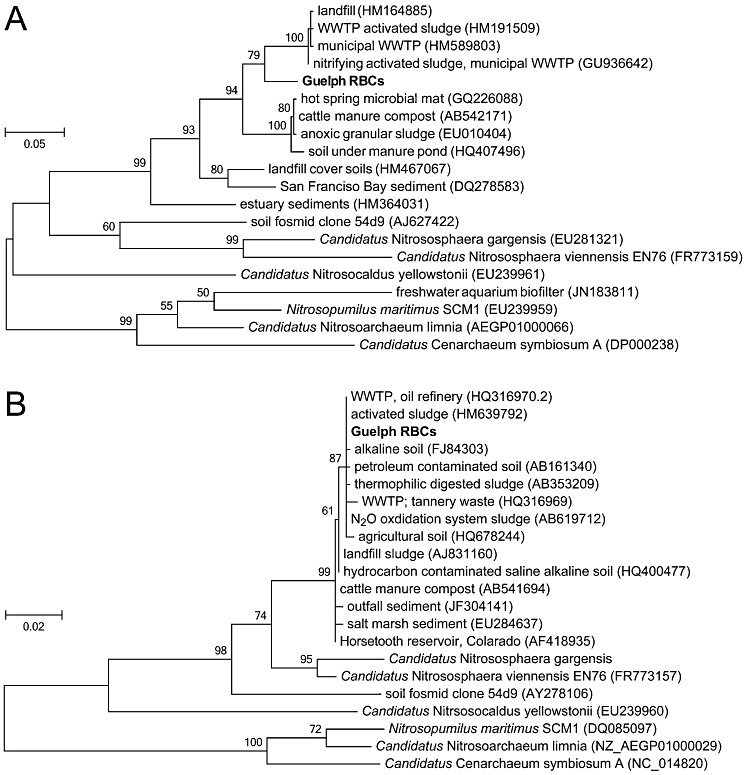
Phylogenetic affiliations of thaumarchaeal *amoA* (A) and 16S rRNA (B) gene sequences retrieved from Guelph WWTP RBCs. Both trees were inferred using the Maximum Likelihood method based on the Tamura–Nei model of sequence evolution. Bootstrap values are located above branches and are based on 500 replicates. Only bootstrap values greater than 50% are indicated on tree. The scale bars represent 5% and 2% nucleotide divergence for A and B respectively.

As described above, DGGE profiles showed simple patterns for archaeal *amoA* and 16S rRNA genes. Representative bands from both treatment trains (NE and SE) from all seasons were excised from gels and sequenced to confirm that populations were identical across all RBC stages, treatment trains and seasons. The resulting *amoA* sequence clustered with environmental sequences derived from other engineered environments, including landfills and activated sludge of municipal WWTPs (Fig. 6A). The *amoA* sequence retrieved from the Guelph RBCs shared a sequence identity of 94% with its most closely related sequences, which each originated from municipal WWTPs (HM589803, GU936642). Similarly, the thaumarchaeal 16S rRNA gene sequence obtained from Guelph WWTP clustered with genes retrieved from high-nutrient environments such as fertilized soils and WWTPs, and clustered distinctly from isolated or enriched AOA representatives (Fig. 6B). This sequence shared 100% sequence identity with sequences retrieved from an industrial WWTP (treating oil refinery waste) (HQ316970), activated sludge from a municipal WWTP (HM639792), and with sludge from a nitrous oxide oxidation system (AB619712).

## Discussion

The present study suggests niche adaptation of AOA to low-ammonia conditions, and identifies a wastewater environment in which AOA are abundant. For each RBC treatment train analysed, AOA populations increased in abundance as ammonia decreased along the flowpath (Fig. 2). When all RBCs from all seasons were analysed together, a negative correlation with high statistical significance (*r* = −0.6887, *P* < 0.0001) was observed between ammonium concentration of wastewater and relative abundance of AOA, implying that ammonia availability is an important factor in determining the relative proportions of ammonia-oxidizing populations.

Ammonium concentrations in the SE treatment train were consistently lower than in the NE treatment train, presumably because this train is located farther from the influent source (see Fig. 1C), and nitrification and volatilization may occur in the wastewater en route to the treatment train. Thaumarchaeal *amoA* and 16S rRNA genes were consistently more abundant in the SE treatment train (Fig. 2B), further supporting the inference of niche adaptation of AOA to low-ammonia conditions. Moreover, AOA gene abundances were highest in September, when ammonia concentrations were lowest. We expected AOB *amoA* genes to decrease across a given RBC flowpath in a pattern that was opposite to that observed for thaumarchaeal genes. However, AOB abundances showed no consistent pattern across the RBCs, indicating that ammonia concentrations remained in a range suitable for AOB growth. On the other hand, our data suggest that ammonia concentrations approached ranges suitable for the detected *Thaumarchaeota* towards RBC 8.

In all RBC stages from all seasons, AOA comprised a substantial proportion of the ammonia-oxidizing community (i.e. always greater than 10%), which is an important finding because studies of activated sludge in municipal WWTPs consistently show that AOB dominate ammonia-oxidizing communities, with AOA having low abundance or being absent (Park *et al*., 2006; Wells *et al*., 2009; Jin *et al*., 2010; Zhang *et al*., 2011). These studies have investigated suspended growth treatment systems used for secondary treatment, where high ammonia concentrations likely preclude the growth of AOA. Although two previous studies have suggested that AOA are abundant in aeration basins of municipal WWTPs (Limpiyakorn *et al*., 2010; Kayee *et al*., 2011), both studies used circular plasmids for qPCR standards, a practice that has been shown to cause serious overestimation of absolute gene copy numbers (Hou *et al*., 2010). Therefore, it remains unlikely that AOA are abundant in traditional aeration basin treatment systems.

Although overall abundance patterns between thaumarchaeal *amoA* and 16S rRNA genes were consistent (Fig. 2B), thaumarchaeal 16S rRNA genes were approximately an order of magnitude higher than archaeal *amoA* genes. It is currently unknown whether all *Thaumarchaeota* oxidize ammonia; however, two lines of evidence suggest that all thaumarchaeal populations in the Guelph WWTP RBCs possess *amoA* genes. First, the similarity between thaumarchaeal *amoA* and 16S rRNA gene abundances across a given RBC flowpath is readily apparent (Fig. 2B), which would be unlikely if these genes did not represent the same population. Moreover, DGGE and sequencing of both thaumarchaeal *amoA* and 16S rRNA genes (Figs 5 and 6) suggest that a single detectable thaumarchaeal population inhabits this wastewater treatment system. It is also possible that higher thaumarchaeal 16S rRNA gene copy numbers result from archaeal populations that possess multiple 16S rRNA operons. However, given that all available AOA genomes contain only one 16S rRNA gene (Hallam *et al*., 2006; Walker *et al*., 2010; Blainey *et al*., 2011; Kim *et al*., 2011), it is more likely that low AOA *amoA* numbers result from poor primer matching with template DNA, consistent with previous findings (Konstantinidis *et al*., 2009). Indeed, although thaumarchaeal 16S rRNA genes were readily amplified from all samples, *amoA* genes could not be reliably amplified without use of a degenerate reverse primer and a decreased annealing temperature (data not shown). In this study, we considered archaeal *amoA* genes to be markers for AOA in an attempt to be conservative in our conclusions. However, based on thaumarchaeal 16S rRNA gene abundances, we have likely underestimated the abundance of AOA based on measured *amoA* gene copies, and therefore potential archaeal contributions to ammonia oxidation in this RBC treatment system.

In addition to qPCR data, this study quantified crenarchaeol as an independent metric of AOA abundance. Crenarchaeol is a glycerol dialkyl glycerol tetraether (GDGT) lipid that serves as a biomarker for AOA in ecological studies and has been detected in isolated and enriched AOA, including *N. maritimus* SCM1 (Schouten *et al*., 2008), *Candidatus* Nitrosocaldus yellowstonii (de la Torre *et al*., 2008), *Candidatus* Nitrososphaera gargensis (Pitcher *et al*., 2010) and *Candidatus* Nitrosoarchaeum limnia (Pitcher *et al*., 2011a). Intact polar lipids are found in cell membranes of living organisms, and are therefore considered to be biomarkers for viable cells. Upon cell death, polar head groups are readily hydrolysed, resulting in CLs, which are indicative of fossilized biomass. Lipid analyses are valuable and comparatively unbiased because of the lack of an amplification step. Indeed, Pitcher and colleagues (2011a) found strong congruence between crenarchaeol IPLs and the abundance and expression of thaumarchaeotal 16S rRNA in the Arabian Sea. In a representative full-length treatment train (June, NE train), both CL and IPL crenarchaeol abundances increased across the RBC flowpath (Fig. 4A). In addition, both core and IPL-derived crenarchaeol was higher in RBC 8 than RBC 1 for all treatment trains sampled (Fig. 4B). These data corroborate quantitative PCR results, and the high proportion of IPL-derived crenarchaeol suggests that the *Thaumarchaea* detected in this system represent viable cells, which are most likely contributing to ammonia oxidation. A few seasonal discrepancies were observed between lipid and genetic data. For example, crenarchaeol abundances were highest in June biofilm samples, whereas thaumarchaeal gene abundances were highest in September biofilm samples. However, direct comparison of genetic and lipid data can be problematic because measured gene abundances are relative values (i.e. copies per nanogram of genomic DNA), whereas lipid measurements represent absolute abundances (i.e. microgram of crenarchaeol per gram of biofilm). Taken together, the same overall trend is supported by both lipid and genetic data.

Although ammonium concentration correlated with AOA abundances, it had no observable effects on AOA diversity or community composition within the ammonia concentrations measured here (∼30–400 µg l^−1^). Thaumarchaeal *amoA* and 16S rRNA profiles contained one dominant band regardless of RBC stage, treatment train or season. Phylogenetic analyses of thaumarchaeal *amoA* and 16S rRNA genes retrieved from this RBC system demonstrated that archaeal sequences clustered with environmental sequences derived from other relatively high-ammonia environments, and share low homology with sequences from enriched or isolated AOA (Fig. 6). This finding may imply that lineages of AOA exist that are adapted to environments with varying nutrient status.

A variety of laboratory studies have identified ammonia as a key environmental parameter for determining relative abundances or activity of AOA and AOB, particularly in soil environments (Di *et al*., 2010; Taylor *et al*., 2010; Verhamme *et al*., 2011). In addition, laboratory incubations of soils have indicated that AOB are metabolically dominant following ammonium amendment (Jia and Conrad, 2009). The present study has taken advantage of an ammonia gradient created by existing wastewater treatment infrastructure and is unique because it provides a practical example of a phenomenon previously only identified through laboratory experiments.

To our knowledge, four previous studies have documented a relationship between ammonium availability and relative abundances of AOA and AOB in freshwater environments. Herrmann and colleagues (2011) demonstrated an increased ratio of AOB : AOA in simulated creek ecosystems amended with ammonia. In addition, Limpiyakorn and colleagues (2010) suggested ammonia as a potential variable in determining the relative abundances of AOA and AOB in municipal WWTPs, but this study produced no statistically significant correlations. A survey of Bangkok WWTPs reported a negative correlation between AOA *amoA* gene abundance in activated sludge and effluent ammonium concentrations (Kayee *et al*., 2011), but a similar correlation was not found with influent ammonium concentrations. A correlation was also observed between AOA : AOB ratios and ammonia concentration in freshwater aquarium biofilters (Sauder *et al*., 2011).

A recent study of municipal and industrial WWTPs found that AOA genes could rarely be detected in municipal WWTPs, but that in certain industrial WWTPs, thaumarchaeal *amoA* genes outnumbered bacterial *amoA* genes by up to four orders of magnitude (Mussmann *et al*., 2011). In one of these industrial plants, it was shown that the present thaumarchaea are not obligate chemolithoautotrophs, but instead may be using organic carbon for mixotrophic or heterotrophic metabolism. Although all currently cultured or enriched *amoA*-encoding thaumarchaeota oxidize ammonia (Könneke *et al*., 2005; Hatzenpichler *et al*., 2008; de la Torre *et al*., 2008; Tourna *et al*., 2011), this study by Mussmann and colleagues (2011) calls into question whether all *Thaumarchaeota* possessing *amoA* genes mediate ammonia oxidation. Therefore, we acknowledge the possibility that the *Thaumarchaeota* detected in the Guelph RBCs may be obtaining energy from organic carbon instead of, or in addition to, ammonia. Indeed, the 16S rRNA gene sequence retrieved from these RBCs clustered with clone sequences derived from industrial WWTPs analysed by Mussmann and colleagues (Fig. 6), although these sequences did not originate from the WWTP on which the majority of their study was based. Further activity studies and cultivation attempts will be necessary to confirm the role of *Thaumarchaeota* in tertiary wastewater treatment. Nonetheless, the *Thaumarchaeota* identified in this study demonstrate an adaptation to low-ammonia conditions, whether or not they are strict chemolithoautotrophs.

Because the discovery of AOA is a recent phenomenon, most existing nitrification infrastructure has been designed on the premise that it will host populations of AOB. However, this study suggests that AOA may play a role in ammonia oxidation in low-ammonia biofiltration systems, such as nitrifying RBCs, aquaculture, aquarium biofilters and drinking water treatment. These systems rely on nitrification for ammonia removal, but low ammonia concentrations may preclude AOB from obtaining sufficient energy for survival and growth. Existing studies support the hypothesis that AOA dominate low-ammonia engineered systems such as groundwater treatment and distribution systems (van der Wielen *et al*., 2009) and granular activated carbon of drinking water treatment plants (Kasuga *et al*., 2010). This may be important when designing biofiltration systems, because AOB and AOA likely vary in a variety of additional ecological adaptations. For example, previous studies have suggested that AOA thrive in low-oxygen conditions (Park *et al*., 2006; 2010). Therefore, the aggressive aeration typically provided in engineered biofilters (that would be beneficial for AOB) may actually interfere with the ability of AOA to oxidize ammonia as efficiently as possible.

The results of this study provide evidence for a low-ammonia niche of AOA within the RBC system in Guelph, Ontario. Future research will determine the extent to which ammonia concentration affects AOA : AOB ratios in additional WWTPs and biofiltration systems associated within additional engineered environments. This study also provides a foundation for future activity, cultivation and genomic analyses for characterizing nitrogen biogeochemistry within engineered freshwater systems.

## Experimental procedures

### Guelph WWTP design, sample collection and water chemistry analyses

The RBCs in the Guelph WWTP were designed for nitrification and are utilized for tertiary treatment following activated sludge treatment in aeration basins, and prior to sand filtration and chlorination (Fig. 1C). The Guelph WWTP features a total of 32 RBC stages arranged in four treatment trains, with each train situated in a tank that is 39.5 m in length, 8.0 m in width, and has a water depth of 1.6 m. Each treatment train consists of eight individual RBC stages, which wastewater passes through serially. The total medium surface area per RBC is 13 750 m^2^, which results in a combined surface area of 440 000 m^2^. Each RBC is approximately 40% submerged in secondary effluent, and continuous rotation at a velocity of 0.8 to 1.3 r.p.m. is driven by air via centrifugal blowers. The average hydraulic detention time across an RBC treatment train is 53 min.

Samples were collected from the Guelph WWTP (Guelph, Ontario, Canada), which is a full-scale municipal WWTP that serves a population of ∼120 000 and treats an average wastewater volume of 42 216 m^3^ per day (based on data from 2010). Samples were collected in February, June and September 2010. February samples were collected from all stages of the NE treatment train (Fig. 1C). In both June and September, all stages of both the NE and SE trains were sampled. Biofilm and RBC-associated wastewater were collected for each RBC stage. Each RBC contains sampling windows, allowing biofilm to be sampled directly from the internal medium surface. Biofilm samples were collected with an ethanol-treated spatula, stored in sterile plastic tubes, and placed on dry ice immediately, where they remained until transfer to −80°C storage. Water samples from each RBC were collected in sterile plastic tubes and stored on ice until return to the laboratory.

Dissolved oxygen (dO_2_) and water temperature were measured *in situ* using an HQ30d digital probe (Hach Company, Loveland, CO, USA). The pH was measured for all water samples using a DELTA 320 pH meter (Mettler Toledo, Mississauga, ON, Canada) directly upon return to the laboratory and prior to freezing. All water samples were then stored at −80°C, except samples used for DOC measurements, which were filtered (0.22-µm syringe) and stored in the dark at 5°C prior to DOC measurements. Dissolved organic carbon was measured using a Dohrman DC-190 High-Temperature TOC Analyser (Rosemount Analytical, Santa Clara, CA, USA). Samples were acidified using 20% phosphoric acid and sparged to remove dissolved inorganic carbon prior to analysis. Nitrate (NO_3_^-^-N) concentrations were measured by ion chromatography using a Dionex ICS-90 (Dionex, Sunnyvale, CA, USA). Nitrite (NO_2_^-^-N) concentrations were measured by colorimetric analysis using a DU 500 UV/Visible spectrophotometer (Beckman Coulter, Brea, CA, USA). All nitrate, nitrite and DOC analyses were performed in the Environmental Geochemistry Laboratory, Department of Earth and Environmental Sciences, University of Waterloo. Ammonium (NH_4_^+^-N) concentrations were determined fluorometrically, as outlined previously (Holmes *et al*., 1999), using a TD 700 fluorometer (Turner Designs, Sunnyvale, CA, USA).

### DNA extraction and quantification

Genomic DNA was extracted from biofilm samples using the PowerSoil DNA Isolation Kit (Mo Bio Laboratories, Carlsbad, CA, USA) as outlined in the manufacturer's instructions. Genomic DNA extracts were visualized on a 1% agarose gel by standard gel electrophoresis and quantified spectrophotometrically using a NanoDrop 2000 (Thermo Scientific, Waltham, MA, USA).

### Quantitative PCR

Quantification of AOA and AOB *amoA* genes used primers CrenamoA23F and a degenerated version of CrenamoA616R (Nicol *et al*., 2008), and amoA-1F and amoA-2R (Rotthauwe *et al*., 1997) respectively. Thaumarchaeal and general bacterial 16S rRNA genes were quantified using primers 771F and 957R (Ochsenreiter *et al*., 2003) and 341F and 518R (Muyzer *et al*., 1993) respectively. All qPCR amplifications were conducted in duplicate on a CFX96 system (Bio-Rad, Hercules, CA, USA). Each reaction volume of 12.6 µl contained 2× iQ SYBR Green Supermix (Bio-Rad), 5 pmol of each primer, 5 µg of bovine serum albumin and 1–10 ng of genomic DNA as template. This template concentration represented a 10-fold dilution of the extracted genomic DNA, and qPCR for serial dilutions of genomic DNA indicated no inhibition at this dilution. For AOA *amoA* genes, the PCR conditions were 95°C for 3 min followed by 35 cycles of 95°C for 30 s, 53°C for 30 s and 72°C for 1 min, with a fluorescence reading following each elongation step. For AOB *amoA* genes, PCR conditions were the same, but with an annealing temperature of 58°C. For both archaeal and bacterial 16S rRNA genes, PCR conditions were the same but with an annealing temperature of 55°C and an elongation time of 30 s. Standard curves were constructed using 10-fold serial dilutions of template DNA of known concentration. For all genes, template DNA consisted of PCR amplicons generated from the same primer pair used for qPCR. For AOA and AOB *amoA* and thaumarchaeal 16S rRNA gene amplicons, the original template source was a freshwater aquarium biofilter (Sauder *et al*., 2011). For bacterial 16S rRNA genes, the original template source was *Escherichia coli* genomic DNA.

Polymerase chain reaction amplification efficiencies ranged from 80.2% to 98.1% and all *R*^2^ values were greater than 0.99. For all amplification reactions, melt curves were performed from 65°C to 95°C with an incremental increase in temperature of 0.5°C. Polymerase chain reaction specificity was verified for all reactions using melt peaks and standard 1% agarose gel electrophoresis.

### Lipid analysis

Biofilm samples were freeze-dried and extracted (3×) using a modified Bligh and Dyer (1959) technique. A solvent mixture of methanol (MeOH) : dichloromethane (DCM) : K phosphate buffer at pH 7.4 (2:1:0.8, v/v/v) was added to the sample in a centrifuge tube and placed in an ultrasonic bath for 10 min. The extract was collected after centrifuging the sample at 2500 r.p.m. for 2 min. Dichloromethane and phosphate buffer were added to the combined extracts to a new volume ratio of 1:1:0.9 (v/v/v) to achieve phase separation. The organic DCM phase and aqueous MeOH/phosphate buffer phase were separated by centrifuging at 2500 r.p.m. for 2 min. The DCM phase, containing the lipids, was passed over extracted cotton to remove possible remaining particles and collected in a glass tube. The aqueous phase was subsequently rinsed twice with DCM, and all cleaned DCM phases were combined and dried under a N_2_ flow and stored at −20°C until analysis.

The extracts were separated into a CL and an IPL fraction over an activated silica column according to Oba and colleagues (2006) and Pitcher and colleagues (2009), except that hexane : ethyl acetate (1:1, v/v) was used to retrieve the CLs, and that MeOH was used to obtain the IPLs. A C_46_ internal GDGT standard (0.1 µg) was added to the CL fraction and an aliquot of the IPL fraction according to Huguet and colleagues (2006), after which the IPL aliquot was subjected to acid hydrolysis to cleave all ether-bound and most of the ester-bound head groups and release their CLs (IPL-derived GDGTs).

Subsequently, the CL and IPL-derived fractions were dissolved in hexane : isopropanol (99:1, v/v), filtered over a 0.45 µm PTFE filter, and concentrated to ∼3 mg ml^−1^ prior to analysis using HPLC/atmospheric pressure chemical ionization–MS on an Agilent 1100 series LC/MSD SL according to Schouten and colleagues (2007), with minor modifications. In short, component separation was achieved with an Alltech Prevail Cyano column (150 mm × 2.1 mm; 3 µm). The GDGTs were eluted isocratically with 90% A and 10% B for 5 min and then with a linear gradient to 16% B for 34 min, where A = hexane and B = hexane : isopropanol (9:1, v/v). The injection volume for all samples was 10 µl. Single ion monitoring of (M + H)^+^-ions was used to detect and quantify the GDGTs. Absolute quantification was performed as described by Huguet and colleagues (2006), in which a typical analytical standard deviation of 5% was reported.

### DGGE and band sequencing

DGGE fingerprinting was performed for AOA and AOB *amoA* genes, as well as thaumarchaeal and general bacterial 16S rRNA genes. DGGE for AOA *amoA* genes was performed as described previously (Tourna *et al*., 2008) with minor modifications. The *amoA* genes were amplified using primers CrenamoA23f and a degenerated version of crenamoA616R, and template concentrations ranged from approximately 0.5 ng to 10 ng per reaction. Polymerase chain reaction conditions were as described previously, except with an annealing temperature of 53°C. Thaumarchaeal 16S rRNA genes were amplified using primers 771F and 957R-GC, with amplification and DGGE conditions as outlined previously (Tourna *et al*., 2008). AOB *amoA* genes were amplified in a nested PCR approach, using primers amoA-1F and amoA-2R, followed by amoA1F-GC and amoA-2R, as outlined by Chu and colleagues (2007). For general bacterial 16S rRNA genes, amplification was performed using primers 341F-GC and 518R, and DGGE was performed as outlined previously (Muyzer *et al*., 1993).

All gels were run at 60°C and 85 V for 900 min, except general bacterial 16S rRNA gels, which were run for 840 min. The DGGE system used was a DGGEK-2401 (C.B.S. Scientific Company, Del Mar, CA, USA) using previously described technical modifications (Green *et al*., 2010). Gels were stained with SYBR green (Invitrogen) for 1 h, then scanned using the Typhoon 9400 Variable Mode Imager (GE Healthcare, Piscataway, NJ, USA) or the PharosFX (Bio-Rad). From the original gel images for each gene fragment analysed, fingerprints were normalized and aligned with GelCompar II (Applied Maths, Austin, TX, USA).

For thaumarchaeal *amoA* and 16S rRNA genes, DGGE bands were excised, amplified (using the above primers and conditions), and sequenced. Amplified DGGE bands were run on a second gel to ensure that each sequenced band corresponded to the original fingerprint. Because DGGE band sequences arising from thaumarchaeal 16S rRNA genes were short (i.e. < 200 bp), a longer thaumarchaeal 16S rRNA gene sequence was obtained with the archaeal primers 21F (DeLong, 1992) and 957R for the purpose of phylogenetic analysis. The longer sequence encompassed the 158 bp region of the corresponding DGGE band sequence; the sequences were identical across this span, indicating that they represented the same AOA population. These DNA sequences have been deposited in GenBank under accession numbers JN695686 and JN695687 for *amoA* and 16S rRNA genes respectively.

Sequences for thaumarchaeal *amoA* and 16S rRNA genes were compared with reference sequences (obtained from GenBank) of enriched or isolated AOA representatives as well as environmental representatives. Sequences were aligned using MUSCLE (Edgar, 2004), and the resulting alignments were cropped so that all sequences spanned the same 483-bp and 762-bp regions for *amoA* and 16S rRNA genes respectively. Evolutionary histories were inferred by using the Maximum Likelihood method based on the Tamura–Nei model of sequence evolution (Tamura and Nei, 1993). The trees shown were those with the highest log likelihood. Bootstrap testing was conducted with 500 replicates. All alignments and phylogenetic analyses were conducted in MEGA5 (Tamura *et al*., 2011).

## Supporting information

**Fig. S1.** Bacterial *amoA* (A) and general bacterial 16S rRNA (B) gene copies in biofilm samples across RBC flowpaths. Error bars represent standard deviations based on technical duplicates; error bars that are not seen are contained within the symbols.

**Fig. S2.** DGGE fingerprints for bacterial and thaumarchaeal *amoA* and 16S rRNA genes across eight serial RBC stages for all sampling seasons and RBC treatment trains.

**Table S1.** Water chemistry data for RBC-associated wastewater.
