# Supplementary material for: Low-ammonia niche of ammonia-oxidizing archaea in rotating biological contactors of a municipal wastewater treatment plant
Source: Environ Microbiol. 2012 Sep;14(9):2589–600. doi: 10.1111/j.1462-2920.2012.02786.x (PMC3466407; doi:10.1111/j.1462-2920.2012.02786.x)

Supporting Information for manuscript:

Low-ammonia niche of ammonia-oxidizing archaea in rotating biological contactors of a municipal wastewater treatment plant

Laura A. Sauder[1], Francien Peterse[2], Stefan Schouten[2], Josh D. Neufeld[1]*

[1] Department of Biology, University of Waterloo, Waterloo, Ontario, Canada

[2] Department of Marine Organic Biogeochemistry, NIOZ Royal Netherlands Institute for Sea Research, Texel, Netherlands

[†] Current address: ETH Zürich, Geological Institute, Zürich, Switzerland

Table S1. Water chemsitry data for RBC-associated wastewater.

Sample	pH	temp. (°C)	DOC (mg L^{-1})	nitrate (mg L^{-1})	nitrite (µg L^{-1})	ammonium (µg L^{-1})	dO_2 (mg L^{-1})
FNER1	7.2	13.1	NA	29.4	375.8	401.9	7.1
FNER2	7.3	12.2	NA	22.5	294.6	320.3	8.0
FNER3	7.4	12.1	NA	28.1	245.9	260.9	8.3
FNER4	7.4	12.6	NA	28.4	186.4	223.8	7.8
FNER5	7.5	12.1	NA	28.9	148.5	147.5	8.9
FNER6	7.4	11.9	NA	28.2	170.1	210.6	8.7
FNER7	7.5	12.5	NA	29.0	148.5	159.3	8.5
FNER8	7.5	12.2	NA	28.5	102.5	106.8	8.7
JNER1	7.4	18.4	10.4	21.2	327.3	252.9	5.9
JNER2	7.4	18.2	10.1	21.5	190.4	135.0	6.9
JNER3	7.4	18.1	10.2	21.7	155.4	128.1	7.3
JNER4	7.5	17.9	10.6	21.1	111.1	112.0	7.6
JNER5	7.5	17.9	10.3	21.5	116.8	89.7	7.7
JNER6	7.5	18.0	10.6	21.7	103.3	81.5	7.4
JNER7	7.5	18.0	10.6	21.4	100.2	79.3	7.5
JNER8	7.6	18.0	10.6	22.0	63.6	45.5	7.8
JSER1	7.4	18.0	11.2	20.9	220.4	147.4	5.5
JSER2	7.4	17.9	10.3	20.9	152.3	103.9	6.7
JSER3	7.4	18.0	10.6	20.9	159.3	105.2	6.5
JSER4	7.4	18.0	9.5	21.5	144.6	95.2	6.8
JSER5	7.4	18.0	10	20.6	115.7	83.3	7.1
JSER6	7.5	17.9	10.3	21.1	107.6	78.4	7.2
JSER7	7.5	18.0	9.8	21.4	84.6	74.2	7.3
JSER8	7.6	18.0	10.3	20.6	72.8	52.3	7.5
SNER1	7.3	21.0	9.3	12.9	91.7	176.6	6.0
SNER2	7.4	21.1	10.3	13.1	66.5	152.6	6.1
SNER3	7.3	21.0	10.7	14.8	56.8	148.7	6.4
SNER4	7.3	21.0	10.0	20.6	65.7	110.2	6.6
SNER5	7.4	20.9	10.0	15.0	51.9	95.4	6.8
SNER6	7.4	20.9	10.7	17.5	46.8	81.6	6.9
SNER7	7.3	21.0	10.6	15.8	27.6	43.1	7.0
SNER8	7.5	21.0	10.2	22.7	38.4	41.2	7.1
SSER1	7.3	21.0	9.6	15.1	51.4	64.5	5.9
SSER2	7.3	21.1	9.3	15.7	39.7	56.6	6.5
SSER3	7.5	21.0	9.0	15.2	35.1	53.2	6.6
SSER4	7.2	21.1	8.8	22.5	43.5	51.9	6.6
SSER5	7.4	21.0	9.1	11.9	27.0	51.1	6.7
SSER6	7.4	21.0	9.1	17.9	28.9	42.9	6.9
SSER7	7.3	21.0	9.1	19.2	43.0	33.8	7.1
SSER8	7.3	21.0	9.1	15.8	21.9	32.8	7.0

DOC: dissolved organic carbon

dO_2: dissolved oxygen

NA: data not available

Figure S1. Bacterial *amoA* (A) and general bacterial 16S rRNA (B) gene copies in biofilm samples across RBC flowpaths. Error bars represent standard deviations based on technical duplicates; error bars that are not seen are contained within the symbols.

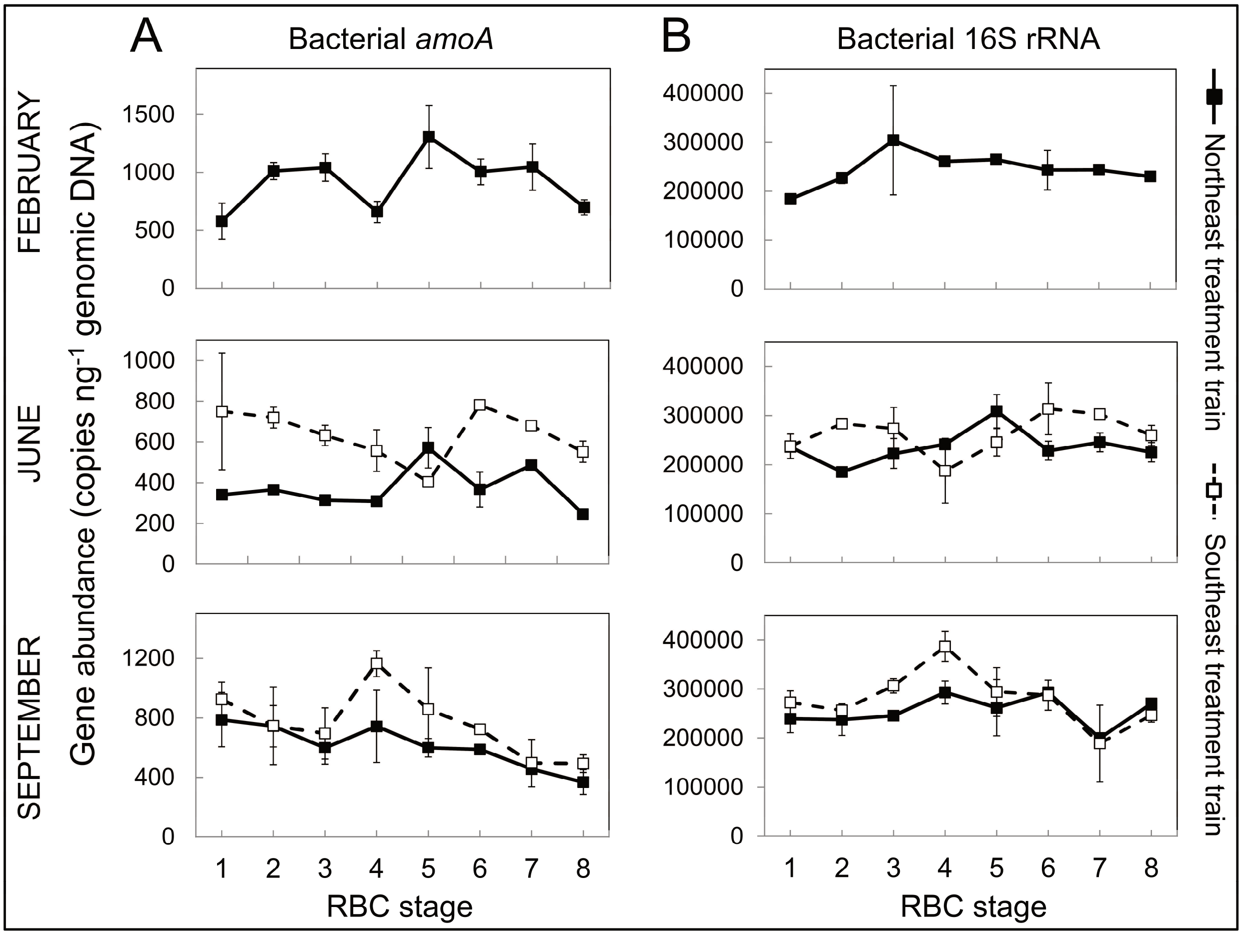

Figure S2. DGGE fingerprints for bacterial and thaumarchaeal *amoA* and 16S rRNA genes across eight serial RBC stages for all sampling seasons and RBC treatment trains.

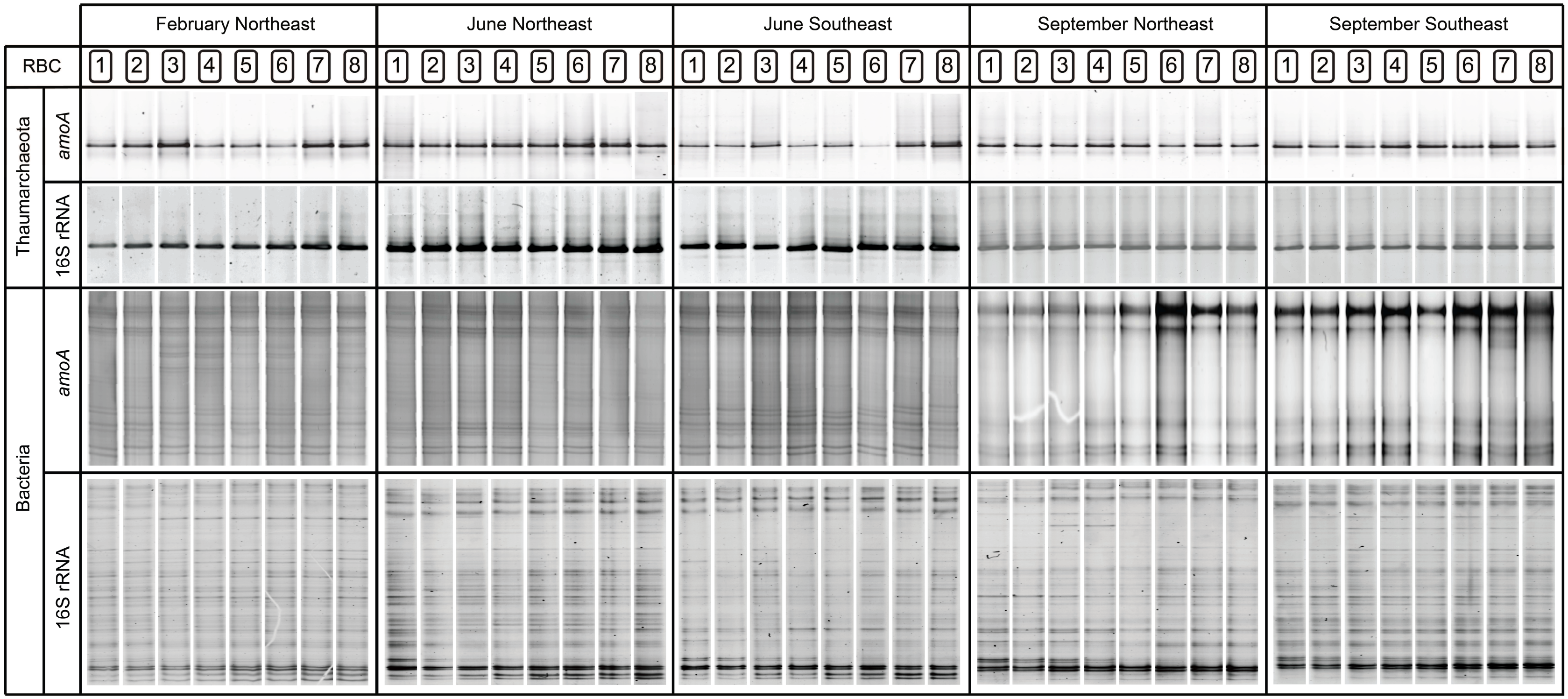

Supplement: Supplementary file 1 [file emi0014-2589-SD1.pdf]
